# Validation of Probabilistic Genotyping Software for Single Cell STR Analysis

**DOI:** 10.3390/genes14030674

**Published:** 2023-03-08

**Authors:** Kaitlin Huffman, Jack Ballantyne

**Affiliations:** 1 Graduate Program in Chemistry, Department of Chemistry, University of Central Florida, Orlando, FL 32816-2366, USA; 2 National Center for Forensic Science, Orlando, FL 32816-2367, USA; 3 Department of Chemistry, University of Central Florida, Orlando, FL 32816-2366, USA

**Keywords:** mixture deconvolution, probabilistic genotyping, single-cell analysis

## Abstract

Probabilistic genotyping (PG) and its associated software has greatly aided in forensic DNA mixture analysis, with it primarily being applied to mixed DNA profiles obtained from bulk cellular extracts. However, these software applications do not always result in probative information about the identity of all donors to said mixtures/extracts. This is primarily due to mixture complexity caused by overlapping alleles and the presence of artifacts and minor donors. One way of reducing mixture complexity is to perform direct single cell subsampling of the bulk mixture prior to genotyping and interpretation. The analysis of low template DNA samples, including from single or few cells, has also benefited from the application of PG methods. With the application of PG, multiple cell subsamples originating from the same donor can be combined into a single analysis using the software replicate analysis function often resulting in full DNA profile donor information. In the present work, we demonstrate how two PG software systems, STRmix^TM^ and EuroForMix, were successfully validated for single or few cell applications.

## 1. Introduction

The application of single/few cell analysis to mixture deconvolution is a growing field in forensic DNA analysis. By analyzing many individual or few cell subsamples collected from a bulk complex DNA mixture, an increase in probative DNA information is often achieved compared to standard DNA mixture approaches (i.e., the bulk homogenization and extraction of a cellular stain followed by PCR amplification, capillary electrophoresis (CE), and probabilistic genotyping (PG)) [1,2,3,4,5,6,7,8,9,10,11,12,13]. In the event of single cell analysis, high quality single source DNA profiles are often obtainable allowing for a significant decrease in the complexity of mixture analysis, since a single recovered cell originates from only one of the several individuals comprising the mixture. To further improve the DNA quantity and, more often than not, the profile quality, multiple cells can be collected within an individual subsample. This improves the chances of recovering a full DNA profile if all of the collected cells originate from the same donor, or a simplified, reduced complexity mixed DNA profile (referred to as a “mini-mixture”) if the cells originated from multiple donors. Even in the instances of these mini-mixtures, profile complexity is often decreased by artificially altering the number of contributors (NOC) or the donor weight ratios compared to the standard bulk mixture.

PG analysis has primarily been applied to bulk DNA extracts. However, these applications do not always result in probative information about the identity of all donors to said mixtures/extracts [14]. This is primarily due to the mixture complexity caused by overlapping alleles, the presence of artifacts, and minor donors [1,2,3,12,15,16,17]. One way of reducing mixture complexity is to perform direct single cell subsampling of the bulk mixture prior to genotyping and interpretation. The analysis of low template DNA samples, including from single or few cells, has also benefited from the application of PG methods. As single cell methodologies have continued to develop, PG applications have now been applied to single or few cell subsamples [2,3,18]. With the application of PG, multiple cell subsamples originating from the same donor can be combined into a single analysis using the software replicate analysis function, often resulting in full profile donor information (i.e., likelihood ratios (LRs) equaling the inverse of the random match probability (RMP) of the donor reference profile) [2].

In the present work, two PG systems that use different models, STRmix^TM^ and EuroForMix (EFM), were validated for single or few cell applications. The validation process was comprised of two phases, the empirical determination of analytically derived parameters that often confound forensic DNA mixture analysis, and then the testing of said parameters to analyze known samples.

## 2. Methods

The direct single cell subsampling (DSCS) methodology used in this study has been previously reported in various publications [1,2,3,18,19,20,21] with a detailed on-line video demonstrating the collection procedure provided in reference [19].

### 2.1. Cell Suspensions/Mixture Samples/Gel-Film Slide Creation

Donor buccal swabs (freshly collected according to procedures approved by the Institutional Review Board for the University of Central Florida or dried and frozen) were used to create cell suspensions by agitating individual swabs in 300 μL of TE−4 (or water/1× PBS depending upon the experiment). The resulting solutions were centrifuged at 300 RCF for 7 min resulting in an epithelial cell pellet. The supernatant was discarded and 300 μL of TE−4 (or water/1× PBS) was used to resuspend each pellet. The Countess™ II FL (ThermoFisher Scientific, Carlsbad, CA, USA) automated cell counter was then used to determine the cell concentration of each cell suspension according to the manufacturer’s recommended protocols. Appropriate volumes of individual donor cell suspensions were mixed to create various mixture samples with the desired donor ratios (i.e., 1:1 2-person mixture, 1:1:1 3-person mixture, 1:1:1:1 4-person mixture, 1:1:1:1:1 5-person mixture, and 1:1:1:1:1:1 6-person mixtures). Samples were stored frozen (4 °C).

Sixty microliters of each mixture was then pipetted onto Gel Film^®^ microscope slides and spread out with a sterile swab. Each slide was stained 1–2 min with Trypan Blue, gently rinsed with nuclease-free water, and air-dried overnight. The Gel Film^®^ microscope slides were created prior to mixture deposition by affixing Gel-Pak^®^ Gel-Film^®^ (WF, ×8 retention level, Hayward, CA, USA) to clean glass microscope slides via the adhesive backing and the clear protective covering was removed.

### 2.2. 3M^TM^ Adhesive Slide Creation 

Prior to cell collection, an adhesive slide reservoir was created by adhering 3M™ adhesive (Allied Electronics, Fort Worth, TX, USA) to a clean glass microscope slide with double-sided tape. The adhesive back was then removed, and the slide stored in a desiccator until needed [19,20].

### 2.3. Cell Recovery

Sample slides were visualized using a Leica M205C stereomicroscope (190–240× magnification) and cells collected directly into 1 μL Prep-n-Go Buffer (ThermoFisher Scientific, Carlsbad, CA, USA) in sterile 0.2 mL PCR flat-cap tubes via a tungsten needle and 3M^TM^ water soluble adhesive. More specifically, the tungsten needle was utilized to obtain a small ball of 3M™ adhesive from the created adhesive slide reservoir. The adhesive-tipped needle was then used to adhere selected cells from the sample slide [19,20]. The needle was then inserted into the lysis containing amplification tube until the 3M™ adhesive was observed to solubilize [19,20].

### 2.4. Direct Lysis/Autosomal Short Tandem Repeat (STR) Amplification of Cells

One to five-cell subsamples were collected directly in 1 μL of Prep-n-Go Buffer (ThermoFisher Scientific, Carlsbad, CA, USA) and incubated at 90 °C @ 20 min; 25 °C @ 15 min. The GlobalFiler^TM^ Express amplification kit (ThermoFisher Scientific, Carlsbad, CA, USA) was then used to amplify the cell subsamples after lysis. The amplification reaction mix consisted of 2 μL master mix and 2 μL primer mix added directly to the 0.2 mL flat cap amp tubes containing the lysed cells. A protocol of 95 °C @ 1 min; 32 cycles: 94 °C @ 3 s, 60 °C @ 30 s; 60 °C @ 8 min; 4 °C @ hold was used. This change to 32 cycles is an increase compared to the 29 cycles used with standard analysis. For positive control samples, 1 μL of a 31.25 pg/μL dilution of 007 control DNA was used. Positive control samples (typically 3–4) and negative control samples (0-cell collections and amplification blanks) were also included in every batch.

### 2.5. Reference Samples/Standard Bulk Mixtures

According to manufacturer recommended protocols, reference profiles and standard bulk mixture samples were extracted using the QIAamp DNA Ivestigator Kit (QIAGEN, Germantown, MD, USA) and quantified using Quantifiler^®^ Duo DNA Quantification kit (ThermoFisher Scientific, Carlsbad, CA, USA) on the Applied Biosystems’ 7500 real-time PCR instrument (ThermoFisher Scientific, Carlsbad, CA, USA). One nanogram of DNA extracts were amplified using the GlobalFiler^TM^ amplification kit (ThermoFisher Scientific, Carlsbad, CA, USA) at 29 PCR cycles. Probabilistic genotyping of donor references profiles and complex equimolar 2-6 person mixtures was conducted using STRmix™ v2.8 and EuroForMix v3.1.0 (Quantitative LR MLE based) according to the known NOC.

### 2.6. PCR Product Detection

One microliter of amplified product was added to a master mix consisting of 9.5 μL Hi-Di formamide (ThermoFisher Scientific, Carlsbad, CA, USA) and 0.5 μL GeneScan™ 600 LIZ™ size standard (ThermoFisher Scientific, Carlsbad, CA, USA). Module J6 (15 s injection, 1.2 kV, 60 °C) was used to inject samples on the 3500 Genetic Analyzer (ThermoFisher Scientific, Carlsbad, CA, USA) using POP-4^TM^ polymer (ThermoFisher Scientific, Carlsbad, CA, USA). GeneMapper^TM^ ID-X v1.6 (ThermoFisher Scientific, Carlsbad, CA, USA) software was used to analyze samples.

Analytical thresholds of Blue: 53, Green: 86, Yellow: 46, Red: 63, and Purple: 63 were determined using Equation (1), analyzing 30 negative control subsamples (i.e., thirty 0-cell subsamples as well as amplification blanks). No stutter filtering was applied within the GeneMapper^TM^ software. However, half-back and double back stutter was manually removed from samples prior to analysis with EuroForMix due to the software only modeling forward and reverse stutter.

(1) AT=2(highest peak−lowest trough)

### 2.7. Optimal Cell Suspension Medium

Single source cell suspensions were created according to Section 2.1 using three different suspension mediums 1× PBS, nuclease-free water, and TE^−4^. One-cell and two-cell subsamples (5×) were collected from each cell suspension type. Box plots were created comparing the allele recovery obtained to each cell number and suspension medium.

### 2.8. Stutter and Drop-In Parameters

STRmix^TM^ specific stutter files were created utilizing 75 subsamples (ranging from 1–5 cells) from 5 donors. For STRmix™, stutter regression files were created according to the implementation and validation guide in which the stutter ratio (SR) was calculated per allele and stutter type using Equation (2).

(2) $$SR=\frac{stutter\;height}{allele\;height}$$

The drop-in rate utilized with both systems (0.0164) was determined utilizing thirty-five negative controls (i.e., 0-cell subsamples or amplification blanks).

### 2.9. DSCS Probabilistic Genotyping (PG)

Probabilistic Genotyping Software STRmix™ v2.8 and EuroForMix v3.1.0 (Quantitative LR MLE based) was validated for use with 1–5 cell subsamples. One-cell samples were analyzed as single source and the log(LR) was reported. Two-cell subsamples were analyzed as either single source or as a two-person mixture depending upon the NOC of the sample, except for instances in which the impact of over/underestimating NOC were examined. When EuroForMix was utilized, all subsamples were modeled with degradation, forward, and reverse stutter unless the model failed, in which case the sample was modeled without degradation. The degradation max parameter in STRmix^TM^ was set to 0.1 (as suggested by STRmix^TM^ support), an increase from the default 0.01 setting used for standard DNA mixture parameters. Population frequencies from the FBI extended Caucasian database and a theta value of 0.01 were used with both systems. With EuroForMix, a drop-in lambda value of 0.01 was utilized as well as 3 optimizations.

STRmix^TM^ tests were conducted to examine the impact of various burn-in accepts (i.e., 5000, 50,000, and 500,000) on LR precision of a single source 3-cell subsample. The STRmix^TM^ Model Maker function was utilized to determine various stutter, amplification, and allele distributions using 161 × −1, −2, −3, −4, or 5-cell subsamples. Subsequent testing was conducted with both systems to determine adequate parameter optimization including tests comparing subsample LRs calculated by hand, and each PG system, and the LR impact of drop-in, inhibition, and degradation. Sensitivity and specificity were tested utilizing 110 × 1 cell subsamples, 81 × 2-cell single source subsamples, 50 × 2-cell mini mixture known donor profiles with a N = 1000 person known non-contributor database provided by STRmix™ support (from their training courses). Additionally, the impact of under (N − 1) and overestimating (N + 1) subsample NOC was examined (n = 35 single source subsamples).

## 3. Results and Discussion

### 3.1. Creating Cell-Suspensions/Mixtures

**Figure 1:**
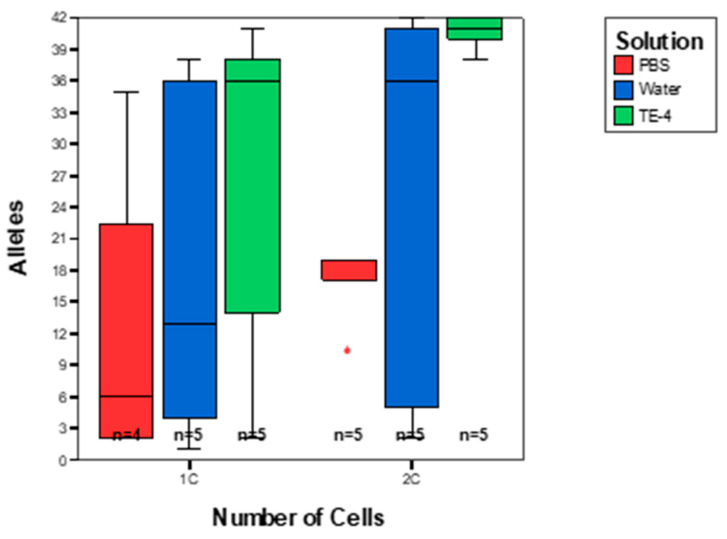
Cell suspensions created in various suspension mediums. 1× PBS (red), nuclease-free water (blue), and TE^−4^ (green). 1C = 1 cell, 2C = 2 cells, n = number of samples analyzed.

Analysis of single cells was posited as an approach to deconvolute complex DNA mixtures by obtaining single source DNA profiles from all donors [1,2,3,4,5,6,7,8,9,10,11,12,13]. To test the applicability of such an approach which we term DSCS (direct single cell subsampling) [1], the creation of known composition complex DNA mixtures was required. One such way to accomplish this is to create individual donor cell suspensions; then, the appropriate concentration of each of the donor’s cells can be combined to create defined mixture ratios. Because the structural integrity of individual cells will depend upon their surrounding environment, different suspension mediums were tested including 1× PBS, water, and TE^−4^. PBS buffer, which although routinely emphasized as a requirement for single cell analysis [22] had a detrimental effect on the resulting DNA profiles. This may be due to the fact that cells analyzed in forensic applications are already dehydrated/lysed and therefore PBS buffer, normally required to maintain a balanced isotonic state between the inside and outside of living, viable cells, may be redundant as well as lacking nuclease protection components. TE^−4^ was therefore chosen as the cell suspension medium of choice due to improved allele recovery compared to 1× PBS and nuclease-free water (Figure 1). This finding is not unsurprising as TE^−4^ does contain nuclease-inhibiting activity. Additionally, aged samples in the form of dried saliva stains and dried stains originating from TE^−4^ cell suspensions were compared in which relatively comparable allele recovery was obtained from both sample types. Although a slight decrease in allele recovery was seen from the cell suspension stains, cell suspensions were concluded to be a viable convenient alternative to saliva to use as a means of preparing defined composition mixtures (Table 1).

### 3.2. Stutter Files

**Figure 2:**
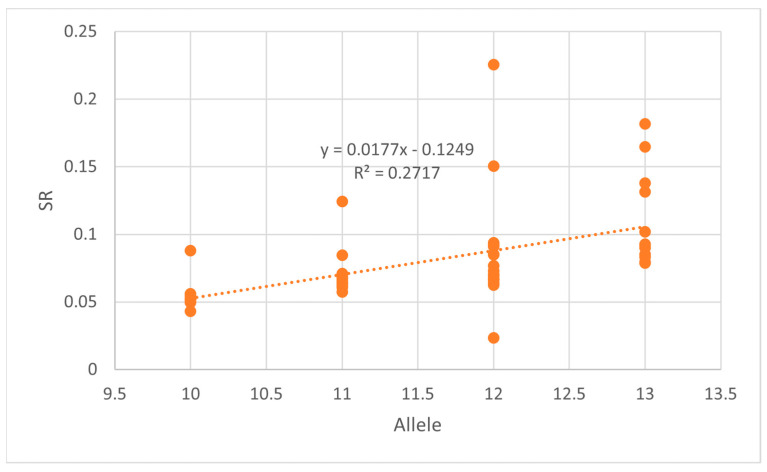
DSCS D16S539 back stutter.

STRmix^TM^ requires optimized stutter parameters for individual loci and allows for modeling of any stutter type. EuroForMix, on the other hand, only models forward and reverse stutter and models all stutter with the same distribution as opposed to per loci and does not require optimization on the part of the user. A graph of SR vs. allele number per loci and stutter type was created (Figure 2). Supplementary Tables S1–S6 provide the slope and intercept (linear stutter) or the average (non-linear stutter) for each locus and stutter type. Due to the presence of elevated stutter, R^2^ values obtained for single cell subsamples are lower than typically observed with standard analysis and high maximum allowable stutter percentages were utilized (back stutter 0.7, forward stutter 0.7, double back stutter 0.3, half-back stutter 0.5, half-forward stutter 0.15, −1.5 repeat stutter 0.15). The stutter distribution parameters were further determined utilizing the STRmix^TM^ Model Maker function.

### 3.3. Saturation Threshold

**Figure 3:**
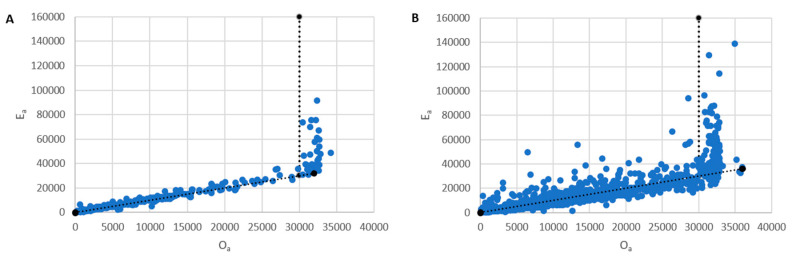
Applied Biosystems^TM^ 3500 Genetic Analyzer saturation threshold (DSCS) (**A**) Standard Analysis (**B**) DSCS. Compared to standard analysis, DSCS samples have a greater discrepancy in observed and expected allele heights due to elevated stutter. Therefore, instrument saturation limits should be determined using standard analysis.

The saturation threshold for the 3500 Genetic Analyzer was determined (30,000 RFUs) by comparing the expected allele height (Ea) and the observed allele height (Oa) from single source samples. The point at which (Ea) and (Oa) begin to consistently diverge is the saturation limit of the instrument. Therefore, any profiles with allele heights > 30,000 RFUs should be diluted and capillary electrophoresis reconducted to prevent the software from incorrectly estimating the Ea in DNA profiles. Because cell subsamples are low template in nature, stochastic effects such as elevated stutter are commonly seen. Therefore, the instrument saturation limit was determined using standard DNA samples (i.e., 25 μL rxn volume amplification of 62.5 pg to 1 ng DNA extracts n = 38) (Figure 3A). Loci with simple repeat structure were utilized (D16S539, CSF1PO, TPOX, D5S818, D13S317, D7S820, and D10S1248). The expected allele heights are determined using the back stutter regression lines determined when creating stutter files for each loci and Equation (3).

(3) $$Ea=\frac{stutter\;peak\;height}{stutter\;ratio}$$

For example, by using the DSCS stutter regression line for D16S539 (𝑆𝑅 = (0.0177 × 𝐴𝑙𝑙𝑒𝑙𝑒 𝑛𝑢𝑚𝑏𝑒𝑟) − 0.1249) (Figure 2), the stutter ratio expected for allele 11 at D16S539 is determined to be SR= 0.0698. However, due to the aforementioned elevated stutter exhibited with DSCS subsamples (n = 189), divergence in the Ea and Oa for our DSCS subsamples is occasionally still seen even below the 30,000 RFUs threshold (Figure 3B).

### 3.4. Drop-In

Drop-in can be described as 1–2 low-level non-reproducible peaks present within a DNA profile [23]. However, because of the low template nature of single cell analysis, drop-in peaks with DSCS samples may have higher peak heights than seen with standard analysis methods. Therefore, a drop-in cap of 30,000 RFUs was used with STRmix^TM^ to allow for any height allele to be considered as possible drop-in. However, re-evaluation of all single-cell subsamples analyzed within this DSCS PG validation as well as subsequent experiments [2,3,18] indicated the highest drop-in peak observed was around 4800 RFU, indicating a drop-in cap of 5000 RFU may be more appropriate and may prevent the software from falsely identifying true allele peaks as potential drop-in. To determine the rate of drop-in occurrences, thirty-five negative controls (i.e., zero-cell subsamples or amplification blanks) were analyzed for the presence of drop-in alleles using Equation (4).

(4) $$Drop\;in\;rate=\frac{number\;of\;drop\;in\;events}{number\;of\;loci\;scored\times number\;of\;samples}$$

The drop-in data did not fit a gamma distribution and limited drop-in data were available. Therefore, a uniform distribution was utilized with STRmix^TM^. The drop-in rate was determined to be 0.0164 (Table 2). The same drop-in rate was used for EuroForMix analysis with the default lambda of 0.01.

### 3.5. Model Maker Results

**Figure 4:**
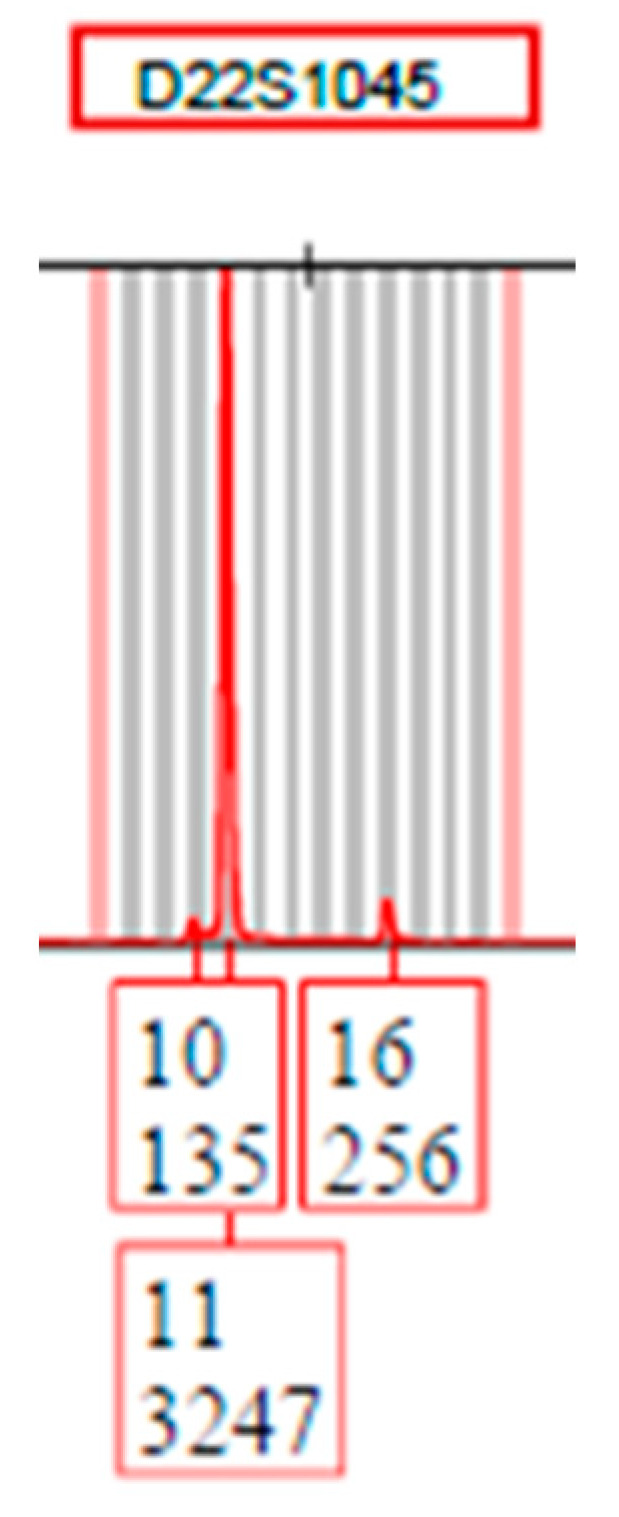
Extreme peak height imbalance possible with single cell analysis illustrated with the locus D22S1045. The known genotype of the cell donor is 11, 16 (10 = back stutter).

Utilization of the STRmix^TM^ software for sample analysis requires creation of a specific protocol-based statistical model that includes the determination of various empirically determined parameter variance probability distribution plots (i.e., stutter, allele, and locus specific amplification efficiency (LSAE)). This requires utilization of the Model Maker function in which single source subsamples are used. It is important for samples utilized within Model Maker to be consistent with the type and quality of samples expected to be encountered in case work. If pristine quality samples were used, then the system may not be able to account for the extreme heterozygous peak height imbalance that is commonly seen with single cell analysis due to the low template nature of the samples (Figure 4). Because of this, 161 × −1, −2, −3, −4, or 5-cell subsamples comprised of fresh buccal cells as well as cells collected from cell suspension mixtures were utilized. The EFM software does not utilize a Model Maker type function.

**Figure 5:**
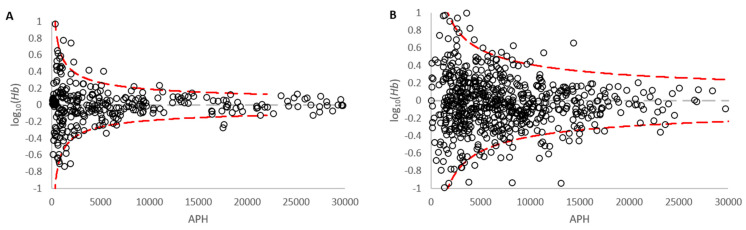
DSCS peak height variability (**A**) Standard Analysis (**B**) DSCS. DSCS results in a broader distribution due to the increased variance/peak height imbalance due to low template effects.

Model Maker performance DSCS versus standard bulk analysis was compared by plotting the log(Hb) vs. Average Peak Height of single source bulk or single cell samples (Figure 5) with the dashed red lines indicating the 95% bounds (+/− 2 standard deviation of the mean) calculated using Equation (5).

(5) $$\pm \sqrt{2}\times 1.96\sqrt{\frac{C^{2}}{APH}}$$

*C^2^* = the 50th percentile of the allelic peak height variance

Utilizing a normal distribution, 87.6% of the single source cell data fell within the 95% bounds. Ideally ≥95% of the data would have been encapsulated within the 95% bounds. A comparison of Figure 5A and B shows that the 95% bounds for the DSCS samples are much broader than standard analysis parameters to account for the low template and stochastic effects seen with single cells (i.e., the extreme peak height imbalance).

### 3.6. Post Burn-In Accepts

**Figure 6:**
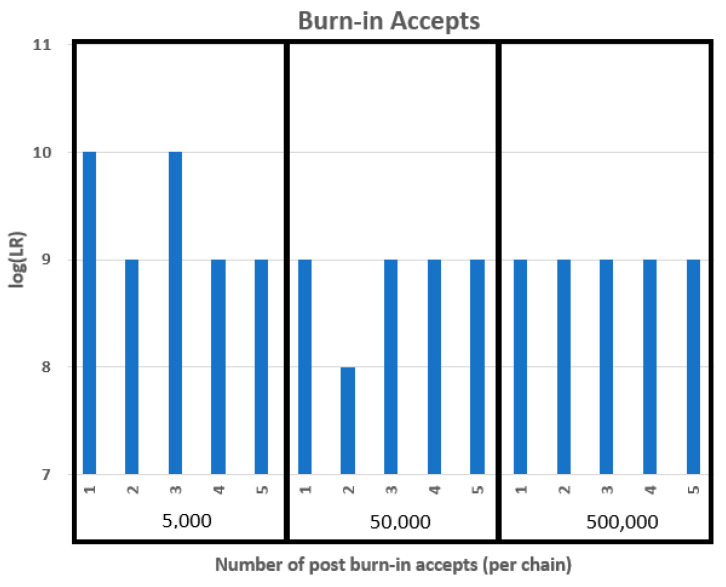
DSCS post burn-in accepts.

With STRmix^TM^, various post burn-in accepts (5000, 50,000, and 500,000) were tested 5× with a single source three-cell subsample (Figure 6). As the number of burn-in accepts increases, so does the time required for analysis as well as the precision of the LR results. STRmix^TM^ default setting is 50,000 burn-in accepts. However, when analyzing single cell subsamples, the most consistent log (LR) results occurred at 500,000 post burn-in accepts.

### 3.7. PG Test of Optimized Parameters

To test the accuracy of the optimized PG parameters, a three-cell subsample (“S5-3C-1”) was analyzed with both PG software systems and the log(LR) was reported. The sample was then artificially degraded by decreasing the peak heights of high molecular weight alleles by 80% and low molecular weight alleles by 5%. An inhibited version of the sample in which the alleles at loci D22S1045, D21S11, D13S317, and D2S1338 were artificially inhibited by decreasing their peak heights by 40% was analyzed. The sample was also tested in which a drop-in eight allele with 15,000 RFUs was artificially added at loci D5S818. With EFM, the same log(LR) results were obtained for all four profiles (i.e., original, with drop in, inhibited, degraded) (EFM Table 3). A figure showing a single dye channel of these artificially created samples was previously published in [2] along with the STRmix^TM^ results demonstrating the sample log(LR) for each of the four sample state scenarios (i.e., log(LR) = 27).

**Figure 7:**
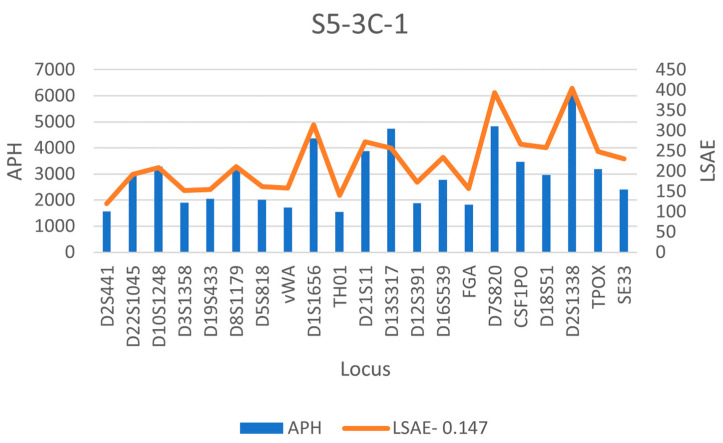
DSCS (3 cell subsample) inter-locus peak height variance.

**Figure 8:**
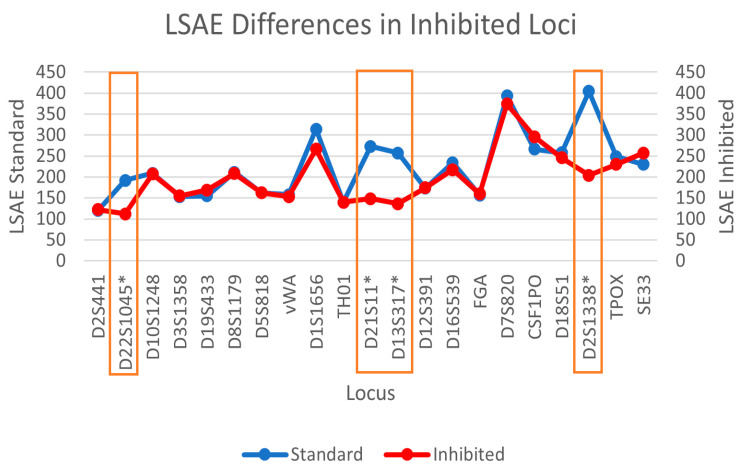
DSCS (3 cell subsample) decrease in locus specific amplification efficiency at inhibited loci (boxed and asterisked).

**Figure 9:**
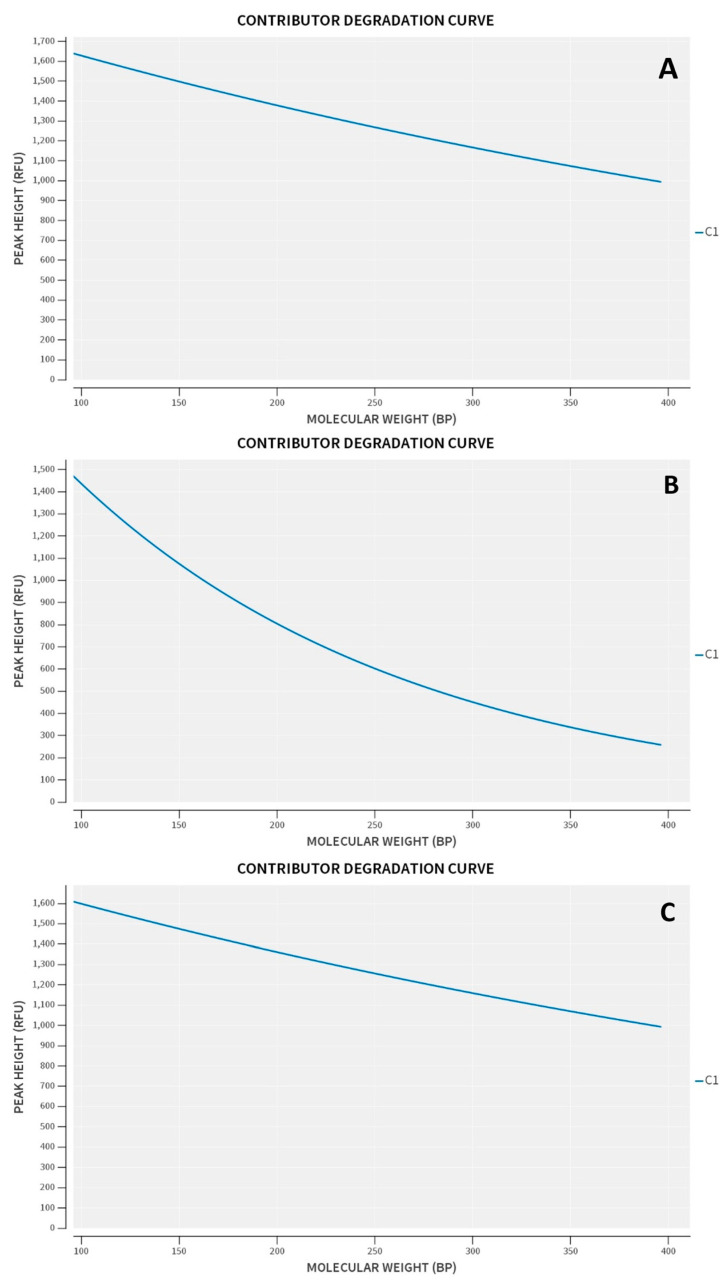
DSCS (3 cell subsample, S3-5C-1) degradation curves. Regular sample (**A**), Degraded sample (**B**), Inhibited sample (**C**).

With STRmix^TM^, the LSAE and APH for the subsample S5-3C-1 are shown in Figure 7 according to increasing molecular weight of the STR loci, indicating that similar trends are seen with the two different parameters. EFM, on the other hand, does not allow for the possibility of different loci having different amplification efficiencies. A comparison of the LSAE obtained for the regular sample and the inhibited sample is then shown in Figure 8 where a decrease in the amplification efficiency trend is obtained for the artificially inhibited loci. The degradation curves obtained from STRmix^TM^, are provided in Figure 9 for the regular sample, degraded sample, and inhibited sample, indicating that only the artificially degraded sample exhibited a characteristic degradation curve that indicated the presence of degradation.

### 3.8. Comparison of PG LR to Hand Calculated LR

The log (LR) for a full single source three-cell profile was calculated by hand and with the PG systems STRmix^TM^ and EuroForMix demonstrating concordant results (i.e., Hand: 27.45, STRmix^TM^ 27.54, and EFM 27.55). The equations utilized to calculate the loci LRs are listed in Equations (6) and (7) [24,25]. The total log(LR) for each method indicates similar results.

(6) $$Heterozygous\;loci=\frac{2\left[\theta +\left(1-\theta \right)p_{i}\right]\left[\theta +\left(1-\theta \right)p_{j}\right]}{\left(1+\theta \right)\left(1+2\theta \right)}$$

(7) $$Homozygous\;loci=\frac{\left[3\theta +\left(1-\theta \right)p_{i}\right]\left[2\theta +\left(1-\theta \right)p_{i}\right]}{\left(1+\theta \right)\left(1+2\theta \right)}$$

Fst = *θ* = 0.01

*p_i_* = allele frequency for allele *i*

*p_j_* = allele frequency for allele *j*

### 3.9. Sensitivity/Specificity

The sensitivity and specificity of the PG systems for DSCS was tested by analyzing 110 × 1 cell subsamples, 81 × 2-cell single source subsamples, 50 × 2-cell mini mixtures samples. With regards to sensitivity (i.e., true positive rate), each subsample was compared (within the PG software) to the known reference DNA profile and a likelihood ratio was reported indicating a strength of inclusion for that known donor. Any instance in which a log (LR) > 0 was obtained was considered inclusionary. A second threshold of log (LR) = 6 was also examined as advantageous matches of unrelated non-donors were not seen when log (LR)s ≥ 6 were achieved. A previous publication [2] has reported the STRmix^TM^ DSCS specificity obtained using these two thresholds. The EFM specificity is reported in Table 4. Replicate refers to ≤6 cell subsamples originating from the same donor probabilistically combined (via a joint probability function) to produce a single LR. As seen in the table and previous publication, utilization of the replicate analysis function resulted in 100% of replicate samples returning inclusionary LRs with both PG systems, while replicate log(LR)s ≥ 6 occurred in 94%, 93%, and 86% of STRmix^TM^ 1-cell, single source 2-cell, and mixed 2-cell subsamples, respectively [2]. A more detailed discussion of replicate analysis is provided in Section 3.11.

**Figure 10:**
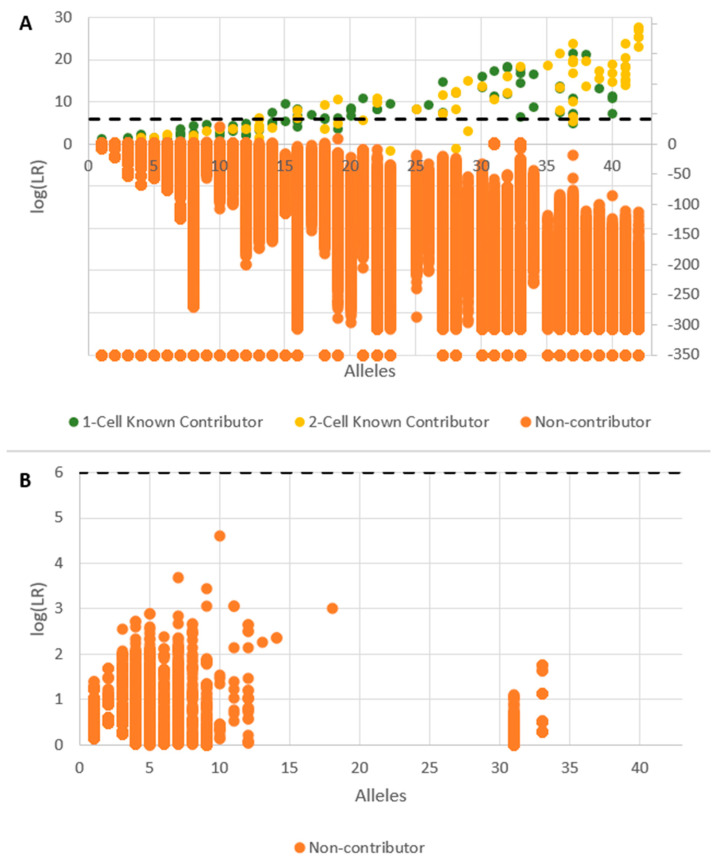
Sensitivity and specificity of DSCS (EFM) analysis with single source subsamples. LR = 0 plotted as −350. Green circles (1-cell known contributor), yellow circles (2-cell known contributor), orange circles (non-contributor). Total results (**A**); False positives (i.e., log (LR) > 0) (**B**).

In order to test the specificity (i.e., true negative rate) of the cell subsamples, a 1000 person known non-contributor database was utilized to test for false positives. False positives occurred any time an inclusionary LR (i.e., log(LR) > 0) was obtained for a known non-donor. So, for every subsample analyzed, 1000 known non-contributor profiles were substituted in the H_1_ position of the likelihood ratio. The same previous publication [2] has reported the results for STRmix^TM^ in which the log (LR) values for known donor subsamples are plotted with respect to the number of alleles detected. The EFM results are provided in Figure 10 where each single source subsample returns a single LR for the known contributor (green or yellow) and 1000 LRs for the non-contributors (orange). Likelihood ratios of 0 were plotted as a log(LR) = −350.

**Figure 11:**
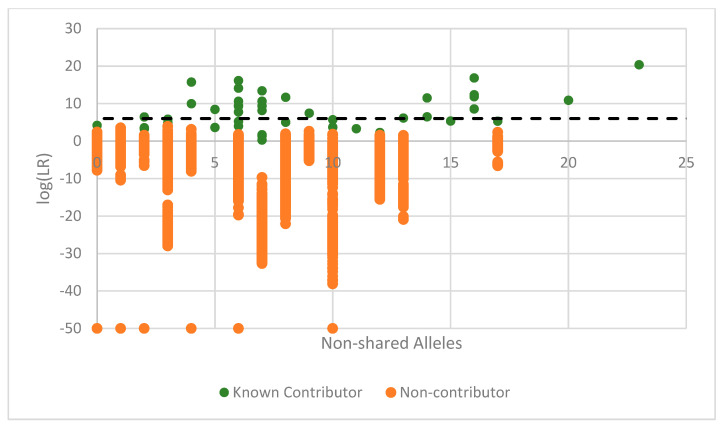
Specificity of DSCS (EFM) mini-mixture analysis. EuroForMix; Single source subsamples. LR = 0 plotted as −50. Green circles (known contributors), orange circles (non-contributor).

As allele count increased, the log (LR) attained for known donors increased while the log(LR)s achieved for non-donors decreased. The majority of false positives occurred at log (LR)s = 1–3, indicative of ‘uninformative’ or ‘limited support’ [26,27]. However, when a 10^6^ LR threshold (dashed line) was utilized, non-contributor false positives were not seen above this measure, and when allele counts exceeded 15, there was generally good separation between known and non-donors. The (LR)s achieved from 2-cell mini mixture subsamples were also analyzed with no false positives being seen at or above the log(LR) = 6 threshold (Figure 11).

### 3.10. Number of Contributors

**Figure 12:**
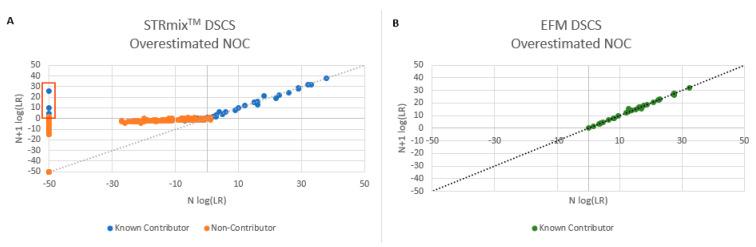
Overestimating the number of contributors to single source DSCS subsamples. STRmix^TM^ (**A**); EuroForMix (**B**); Note: Non-contributor profiles were not run with EFM. LR = 0 plotted as −50.

**Figure 13:**
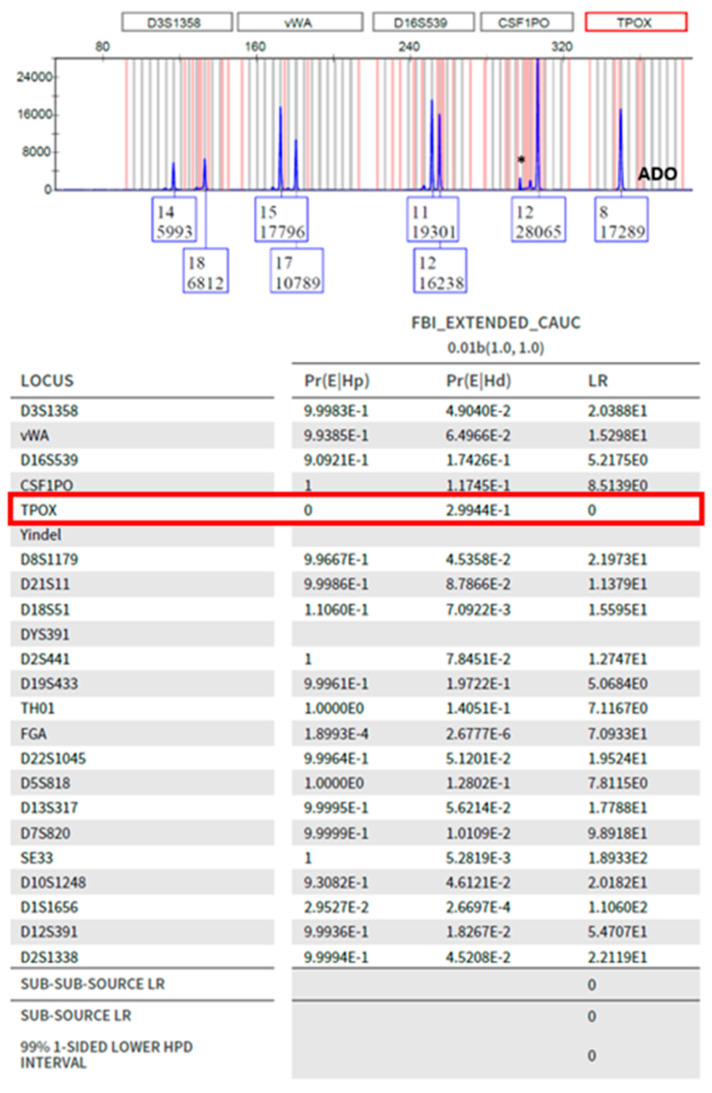
Drop-out from a single heterozygous TPOX allele. ADO = allele drop-out; * = pull-up. Stutter was retained for the sample when analyzed with PG.

The impact of overestimating the number of contributors (N + 1) with DSCS samples was determined by analyzing the 35 single source subsamples (Figure 12). For the majority of samples, comparable results were obtained when analyzing samples according to the correct NOC as well as N + 1 (as indicated by the blue (STRmix^TM^) and green (EFM) data points along the y = x line). With STRmix^TM^, three subsamples returned an LR of zero when analyzed as N = 1. However, when ran as N + 1 (i.e., N = 2) high LRs were achieved (shown boxed in Figure 12). These were samples with drop-out of a heterozygous allele that resulted in an LR = 0 at a specific locus (e.g., Figure 13). By increasing the NOC, the system seems to account for the possibility of this drop-out where it did not appear when ran as N = 1. Misidentifying the number of contributors by N + 1 does not appear to negatively impact analysis.

**Figure 14:**
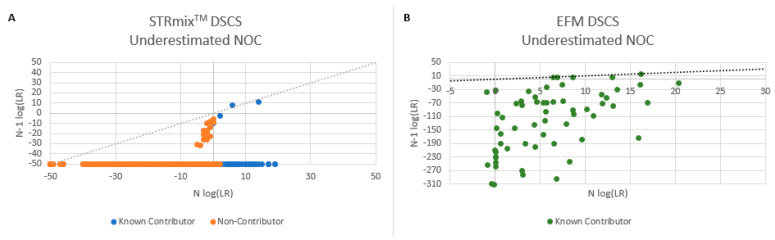
Underestimating the number of contributors to 2-cell DSCS mini-mixtures. (**A**) STRmix^TM^; (**B**) EuroForMix; Note: Non-contributor profiles were not run with EFM. LR = 0 plotted as −50.

The impact of underestimating the number of contributors (N − 1) with DSCS samples was determined by analyzing the 35× 2-cell mini-mixture subsamples (Figure 14). As indicated by the blue and green data points along the y=x line, underestimating the NOC has a detrimental impact on analysis as true donors may be falsely excluded. This is not entirely surprising as underestimating the NOC in these instances would be misidentifying a mini-mixture as a single source, which would not occur in most instances. Non-contributor profiles were not analyzed with EuroForMix due to the slow speed of the database search function. The NOC estimation trends observed correlate to those seen with previous PG studies [28].

### 3.11. Replicates

**Figure 15:**
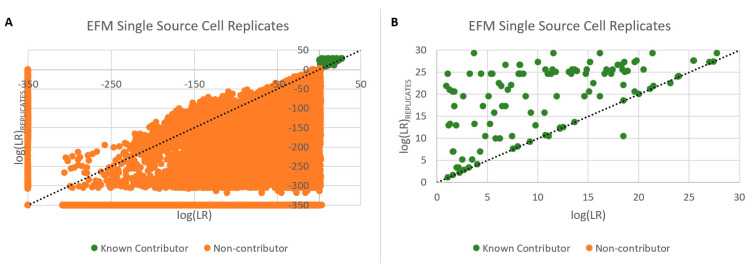
Specificity improvement of DSCS (EFM) with replicate analysis. Known contributors above the diagonal (i.e., y = x) represent improved LR recovery from replicate analysis. Single Source Cell Replicates. LR = 0 plotted as −350. Green circles (known contributors); orange circles (non-contributors). (**A**) All samples. (**B**) Detail showing log(LR) > 0 samples.

**Figure 16:**
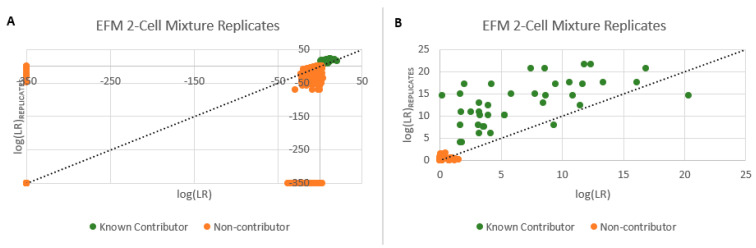
Specificity of DSCS (EFM) mini-mixture analysis. LR = 0 plotted as −350. Green circles (known contributors), orange circles (non-contributor) (**A**) All samples. (**B**) Detail showing log(LR) > 0 samples.

Utilizing the replicate analysis function of PG systems allows for the combination of multiple samplings originating from the same bulk sample into a single analysis. For standard bulk analysis, this takes the form of multiple amplifications of the same original extract [29]. With respect to low template DNA, this has been shown to result in greater profile recovery for known donors as opposed to a single amplification [30] and differentiates robust signals from un-replicated artifacts, thus decreasing stochastic effects [31]. The replicate analysis capability of the PG systems was therefore evaluated for use with single or few cell subsamples. Up to 6 cell subsamples originating from the same donor and collected from complex 2–6 person mixtures were utilized with replicate analysis with an average of 4 subsamples (±1) used. An initial screening of these subsamples was conducted in which samples that provided an inclusionary log(LR) (i.e., log(LR) > 1) for a specific donor were considered for use. An alternative approach to cluster individual subsamples by donor may be to utilize the mixture to mixture and common donor applications in the DBLR software [32,33], or employ classic clustering algorithms such as K-means or EM [34]. A comparison of the log(LR)s obtained from individual subsamples compared to replicate analysis is provided in Figure 15 (single source) and Figure 16 (two-cell mini-mixtures). In nearly all instances, replicate analysis resulted in improved LRs compared to individual subsamples as indicated by the green datapoints above the dashed line (y = x), while the false positives orange data points decreased. Occasionally, replicate analysis with EFM resulted in a failed analysis presumably due to modeling issues arising from subsamples with differing degrees of degradation. However, the newly developed EuroForMix extension, EFMrep [35], prevented these failed investigations. Similar results were obtained with STRmix^TM^ [2].

Because the aim of direct single cell subsampling is to obtain single source (or less complex mini-mixtures) from a bulk mixture, the PG system’s accuracy was tested by intentionally misclassifying a subsample for replicate analysis. Single source and mini-mixture subsamples with non-inclusionary LRs (LR < 1) for the true donor(s) (i.e., incorrect donor for the replicate grouping) were added to the sample subset used for replicate analysis. Poor quality profiles (i.e., ~4/42 alleles) had relatively no impact on the replicate log(LR) obtained. However, as profile quality increased (by improved allele recovery), a significant decline occurred in the log(LR)s attained (i.e., LRs < 0) (Table 5). For mini-mixtures specifically, inclusionary LRs were occasionally still seen for the true donors when a misclassified subsample was added. However, these LRs were either comparable to the LRs obtained without the misclassified sample or decreased (Table 6). To briefly explain, Table 6 provides the replicate log(LR) obtained when three two-cell mini-mixture subsamples comprising donors S5 and CM31 were utilized. Misclassification was tested by adding an additional ‘incorrect’ subsample to the replicate test (i.e., the three correctly classified S5CM31 mini-mixtures and one misclassified single source subsample) for donors SA10, S3, and S8. Misclassified mini-mixtures were additionally added to the S5CM31 mini-mixture replicate analysis (i.e., the three correctly classified S5CM31 mini-mixtures and one or two misclassified S3SA10 mini-mixtures).

Encouragingly, the data from the EuroForMix analyses using the same samples were in close agreement with STRmix^TM^. Minor discrepancies between the two systems may be partially due to the fact EFM only models back and forward stutter; therefore, any half-back or double back stutter was removed prior to analysis with EFM. Furthermore, all back/forward stutters with EFM are all modeled with the same distribution rather than separately as with STRmix^TM^, and the possibility of different loci having different amplification efficiencies is not considered.

### 3.12. DSCS Deconvolution Compared to Standard Bulk Analysis

**Figure 17:**
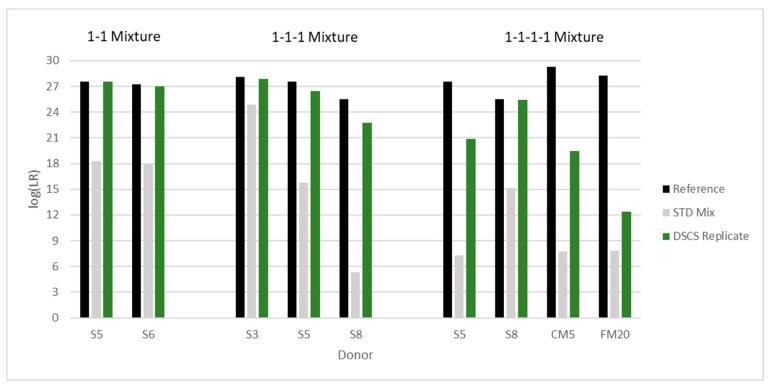
Increased contributor log(LR) recovery in 2–4 person mixtures by DSCS (EFM) (“DSCS Replicate”) compared to standard PG mixture analysis (STD Mix). The “Reference” LR is the reciprocal of the random match probability of the single source DNA profile from the known donor. The alphanumeric characters refer to the individual donors present in the mixture.

**Figure 18:**
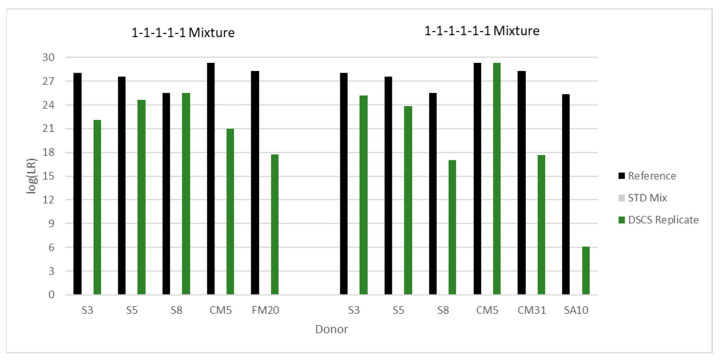
Increased contributor log(LR) recovery in 5 (left panel) and 6 (right panel) person mixtures by DSCS (EFM) (“DSCS Replicate”). Standard PG mixture analysis (“STD Mix”) could not be conducted on these mixtures due to EFM software limitations. The “Reference” LR is the reciprocal of the random match probability of the single source DNA profile from the known donor. The alphanumeric characters refer to the individual donors present in the mixture.

**Figure 19:**
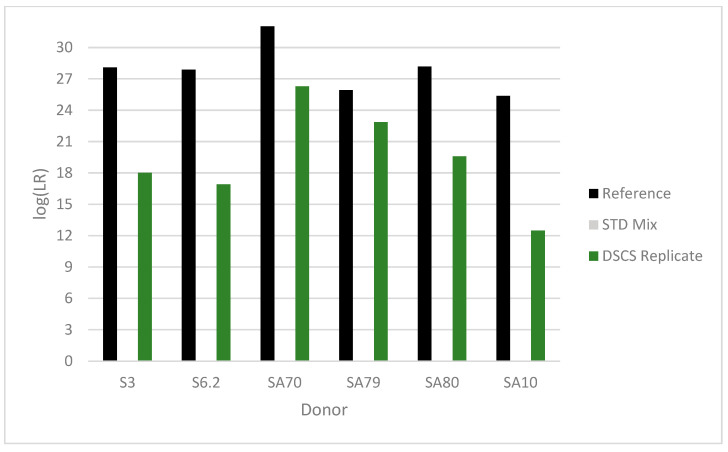
Contributor log (LR) recovery by DSCS (EFM) (“DSCS Replicate”) in an additional 6-person mixture. The “Reference” LR is the reciprocal of the random match probability of the single source DNA profile from the known donor. Standard PG mixture analysis (“STD Mix”) could not be conducted on the mixture due to EFM software limitations. The alphanumeric characters refer to the individual donors present in the mixture.

In a single mixture, the DSCS procedure produces up to six different subsample types with associated LRs from separate putative contributors. These subsample types include one- and two-cell subsamples, two-cell mini-mixture subsamples, one- and two-cell replicates, and mini-mixture replicates. After replicate analysis, the maximum replicate likelihood ratio obtained is reported. Seven equimolar complex mixtures were deconvoluted using this DSCS process. Figure 17, Figure 18 and Figure 19 (EFM) compare the ‘ground-truth’ or maximum possible log(LR) per known donor (i.e., log(1/RMP) from single source reference profiles (black bars)) to the standard analysis results (i.e., log(LR) per donor, where LR = known donor and N-1 unrelated individuals contributing to the mixture vs. N unknown unrelated individuals contributing to the mixture) (grey bars)) and the DSCS replicate log(LR)s per donor (green bars). Improved genotype information was achieved per donor for each mixture with the most significant improvement occurring with the complex five- and six-person mixtures (Figure 18 and Figure 19). Standard analysis was unable to be conducted for these mixtures due to limitations of the software as it is only recommended on mixtures comprising up to four people [36]. The STRmix^TM^ results for these same mixtures have been previously reported [2]. The two different PG software systems returned remarkably similar individual contributor LR values with no significant discordant results. The EFM log(LR) results ranged from 6 to 29, while STRmix^TM^ results ranged from 8 to 28. This represented an additional diagnostic check [37] on the accuracy and reliability of the DSCS method’s performance with the sample set studied.

### 3.13. Upgraded Version of PG Software (STRmix^TM^ Update to Version 2.9.1)

PG software is being continuously updated. It behooves the user to test and evaluate the new version before relying on it to perform additional analyses. Since the initial validation and comparison of the two PG systems reported in this study, an updated version of STRmix^TM^ has been released. This section is included to illustrate to readers how the performance of a new version of PG software (using STRmix^TM^ as an example) can be compared to the one used for previous work. Upgrading to STRmix^TM^ v2.9.1 requires remodeling of the Model Maker parameters [38]. For the same data previously used to model v2.8, the updated v2.9.1 Model Maker parameter results are listed in Supplementary Figure S1. Fifty-five subsamples were then re-analyzed with version 2.9.1 (Supplementary Figure S2) showing there is close agreement in the log(LR)s obtained with versions 2.8 and 2.9.1.

## 4. Conclusions

Two different PG software systems, STRmix^TM^ and EuroForMix, which employ different models to deconvolute forensic DNA mixtures were validated for single cell (and few cell) analysis. While only two software systems were evaluated, it is envisioned that single cell PG analysis should be possible with other creditable PG software systems after appropriate validation. The single cell PG validation process is comparable to those validation studies conducted for standard DNA bulk extract analysis, although there are some notable differences. Differences occur (for STRmix^TM^ specifically) in that rather than utilizing single source DNA extracts spanning profile quality observed within casework (i.e., < 1 ng), 1-, 2-, 3-, 4-, and 5-cell single source subsamples were utilized with the Model Maker function for DSCS validation. Similarly, stutter files are created for the DSCS process using 1-5 cell subsamples. Validation for the use of a higher number of subsampled cells (i.e., >5), is possible; however, a high degree of saturation was observed when >5 cells were used with our specific single cell workflow (i.e., 5 μL rxn volume and 32 cycles). Other marked differences occur in that rather than creating sensitivity and specificity plots according to RFUs, allele count is used instead (e.g., Figure 10 and Figure 11).

While the overall process of validating PG for DSCS analysis is broadly similar to that performed for standard workflows, many of the optimized parameter values are markedly different. For example, the default drop-in cap and rate for STRmix^TM^ v2.8 is 100 RFU and 0.0001, while those used with DSCS are 30,000 RFUs (though this can likely be decreased to 5000 RFUs) and 0.0164. Similarly, due to the low template effects of single cell analysis, an increase in elevated stutter is experienced with DSCS samples, thus resulting in high stutter maximums being utilized (e.g., 0.7 for forward and reverse stutter). This was further exemplified in Figure 3 and indicates that saturation thresholds (which are largely instrument dependent) should be determined using standard samples rather than single cell subsamples. Additionally, an increase in burn-in accepts from 50,000 to 500,000 is recommended for single cell analysis as this allows the software additional time to converge on the proper result.

The biggest challenge with single cell analysis arises due to enhanced allele drop-out and peak height imbalance, especially in the extreme (but not uncommon) case in which a single allele from a heterozygous pair drops out while the sister allele is present at a high RFU (e.g., Figure 13). This can often result in the system adjudging the remaining allele as a homozygous allele especially if a high RFU is observed. This extreme imbalance was exemplified in Figure 5 showing the much broader variance experienced with single cells compared to standard analysis. However, even so, highly probative results are still able to be obtained, particularly when the PG replicate analysis function is utilized, which often results in DNA profile LRs comparable to the inverse of the random match probability for donor reference profiles (i.e., the upper bound of the achievable LRs [30]). The replicate analysis function of the PG software systems is an important component of the described DSCS single cell methodology. This enables the individual genotyping results from single cell subsamples from the same donor to be probabilistically combined (via a joint probability function) to produce a single LR. The effect is to better take into account the stochastic effects of low template DNA (such as in single cells) while at the same time recovering more of the true alleles originating from an individual donor (that would otherwise be lost due to drop out in a single sample). Since the initial validation of the PG software systems, we have modified our direct lysis/PCR strategy. The Casework Direct System (Promega, Madison, WI) [39] has been found to significantly improve the quality of DNA profiles obtained with single cells (i.e., increased number of alleles recovered), prior to their joint use with the PG software replicate analysis functions.

Perhaps one of the largest impediments to labs considering testing and evaluation of single cell PG applications is the cost associated with preparing and analyzing the hundreds of single cell subsamples needed to develop the PG model. While our manual DSCS protocol is significantly cheaper than other automated single cell sub sampling methods, another approach to potentially decrease cost would be to create in silico models of single cell subsamples [34]. Other work with standard PG analysis has demonstrated comparable success from various labs when utilizing general PG parameters as opposed to lab specific parameters for comparable workflows (i.e., same amplification kit, reaction volume, PCR cycle number, and capillary electrophoresis model) [40]. Therefore, it is feasible that a similar approach may be applicable for PG single cell analysis in the future.

## Figures and Tables

**Table 1:** Allele recovery of 1- and 2-cell subsamples collected after 6 months from either a cell suspension or a saliva stain.

	1-Cell Subsamples	2-Cell Subsample
Saliva Stain	25 ± 12	29 ± 10
Cell Suspension	22 ± 12	26 ± 14

**Table 2:** STRmix^TM^ drop-in data/parameters.

Distribution	Drop-In Events	Samples Analyzed	Loci	Samples × Loci	Drop-In Rate	Drop-In Cap
Uniform	11	35	21	735	0.0164	30,000 RFU

**Table 3:** DSCS parameters test.

Sample	EFM Log(LR)
S5-3C-1	27.55
S5-3C-1 drop-in	27.55
S5-3C-1 inhibited	27.55
S5-3C-1 degraded	27.43

**Table 4:** Percentage of subsamples and replicates analyzed with EuroForMix that reach the specified threshold.

Sample Type	Subsample	Replicate	Subsample	Replicate
	log(LR) > 0		log(LR) ≥ 6	
SS 1 cell (s = 100)	87%	100%	45%	94%
SS 2 cell (s = 81)	89%	100%	64%	93%
Mix 2 cell (s = 110)	60%	100%	30%	93%

**Table 5:** Misclassification of single source subsamples for replicate analysis.

Correct Profile S3				Incorrect Profile Included in Replicate (Various Donors)				
Sample	Total Alleles	STRmix^TM^ Replicate log(LR)	EFM Replicate log(LR)	Misclassified Sample	Total Alleles	Alleles That Do Not Match S3	STRmix^TM^ Replicate log(LR) S3	EFM Replicate log(LR) S3
S3-6	19	14	12	CM31	4	2	14	12
S3-9	18			SA10	7	5	0	−20
				S5-24	14	6	−9	−19
				S5-25	21	11	0	−3
				S8	32	19	0	−101

**Table 6:** Misclassification of mini-mixtures for replicate analysis.

Correct Profiles S5/CM31 Mini-Mixture					Incorrect Profile Included in Replicate (Various Donors)							
Sample	STRmix S5	STRmix CM31	EFM S5	EFM CM31	Misclassified Sample	Total Alleles	Alleles That Do Not Match S5	Alleles That Do Not Match CM31	STRmix Replicate log(LR) S5	STRmix Replicate log(LR) CM31	EFM Replicate log(LR) S5	EFM Replicate log(LR)
S5CM31-4	9	16	2	14	SA10	7	5	3	9	16	8	FAIL *
S5CM31-6	1	15	3	16	S3	19	11	13	0	16	−4	15
S5CM31-28	8	10	9	11	S8	32	20	18	0	16	−30	FAIL *
Replicate	10	17	8	18	S3/SA10-17				0	10	−12	15
					S3/SA10-25				4	16	FAIL *	FAIL *
					S3/SA10-17&25				0	5	FAIL *	FAIL *

* Note: FAIL refers to model validation failure likely due to an unintuitive NOC being utilized.

## Data Availability

Requests for additional underlying data can be made to the corresponding author.

## Supplementary Materials

The following supporting information can be downloaded at: https://www.mdpi.com/article/10.3390/genes14030674/s1, Figure S1: STRmix^TM^ v2.9.1 Model Maker; Figure S2: Comparison of STRmixTM v2.8 and v2.9.1 Table S1: Back stutter; Table S2: Forward stutter; Table S3: Double back stutter; Table S4: Half back stutter; Table S5: Half forward stutter; Table S6: 1.5 bp back stutter.

## Abbreviations

DSCS (direct single cell subsampling); EFM (EuroForMix); LR (likelihood ratio); LSAE (Locus Specific Amplification Efficiency); NOC (number of contributors); POI (person-of-interest); PG (probabilistic genotyping); RMP (random match probability); SR (stutter ratio).
