# Supplementary material for: Validation of Probabilistic Genotyping Software for Single Cell STR Analysis
Source: Genes (Basel). 2023 Mar 8;14(3):674. doi: 10.3390/genes14030674 (PMC10048617; doi:10.3390/genes14030674)
Supplement: Supplementary file 1 [file genes-14-00674-s001.zip › genes-2240204-supplementary.pdf]

SUPPLEMENTARY TABLES

Supplementary Table 1. Back stutter

LOCUS	INTERCEPT	SLOPE
D3S1358	-0.0448	0.0100
vWA	-0.0057	0.0058
D16S539	-0.1249	0.0177
CSF1PO	-0.0532	0.0118
TPOX	-0.0428	0.0089
D8S1179	0.0165	0.0054
D21S11	-0.0283	0.0041
D18S51	-0.0862	0.0118
DYS391	0.085321	0
D2S441	0.0221	0.003
D19S433	-0.0191	0.0074
TH01	0.0017	0.0105
FGA	-0.1407	0.0108
D22S1045	-0.2004	0.0209
D5S818	-0.0684	0.0132
S13S317	-0.0565	0.0106
D7S820	-0.0749	0.0149
SE33	0.0624	0.0022
D10S1248	-0.0104	0.0078
D1S1656	-0.0338	0.0099
D12S391	-0.1644	0.0143
D2S1338	-0.0085	0.0059

Supplementary Table 2. Forward stutter

LOCUS	INTERCEPT	SLOPE
D3S1358	0.010963	0
vWA	0.007421	0
D16S539	0.009135	0
CSF1PO	0.013372	0
TPOX	0.003210	0
D8S1179	0.012906	0
D21S11	0.012221	0
D18S51	0.008596	0
D2S441	0.008966	0
D19S433	0.005052	0
TH01	0.006847	0
FGA	0.008583	0
D22S1045	-0.14030	0.0135
D5S818	0.032547	0
D13S317	0.019247	0
D7S820	0.020042	0
SE33	0.007280	0
D10S1248	0.007718	0
D1S1656	0.012350	0
D12S391	0.006117	0
D2S1338	0.028035	0

Supplementary Table 3. Double back stutter

LOCUS	INTERCEPT	SLOPE
D3S1358	-0.0088	0.0013
vWA	0.013366	0
D16S539	0.018792	0
CSF1PO	0.009483	0
TPOX	0.001332	0
D8S1179	0.013969	0
D21S11	0.008009	0
D18S51	-0.0151	0.0015
DYS391	0.010008	0
D2S441	0.003773	0
D19S433	0.009498	0
TH01	0.007088	0
FGA	-0.0306	0.0017
D22S1045	0.009908	0
D5S818	0.004961	0
D13S317	0.004784	0
D7S820	0.009775	0
SE33	0.0005	0.0003
D10S1248	0.009478	0
D1S1656	-0.0072	0.0012
D12S391	0.013393	0

Supplementary Table 4. Half back stutter

LOCUS	INTERCEPT	SLOPE
SE33	0.047681	0
D1S1656	0.027844	0

Supplementary Table 5. Half forward stutter

LOCUS	INTERCEPT	SLOPE
SE33	0.007651	0
D1S1656	0.020004	0

Supplementary Table 6. 1.5 bp back stutter

LOCUS	INTERCEPRT	SLOPE
SE33	0.005573	0
D1S1656	0.016909	0

PROBABILITY DISTRIBUTION

	α	β	MODE
Allele Variance c^2	26.343	8.705	220.611
Back Stutter Variance k^2	2.068	47.510	50.741
Forward Stutter Variance k^2	2.517	19.898	30.185
Double Back Stutter Variance k^2	8.048	1.999	14.089
-2 bp Stutter Variance k^2	3.781	10.533	29.292
+2 bp Stutter Variance k^2	9.817	17.953	158.292
-6 bp Stutter Variance k^2	8.998	14.841	118.698
LSAE Variance	0.125		

Supplementary Figure 1. STRmix^{TM} v2.9.1 Model Maker

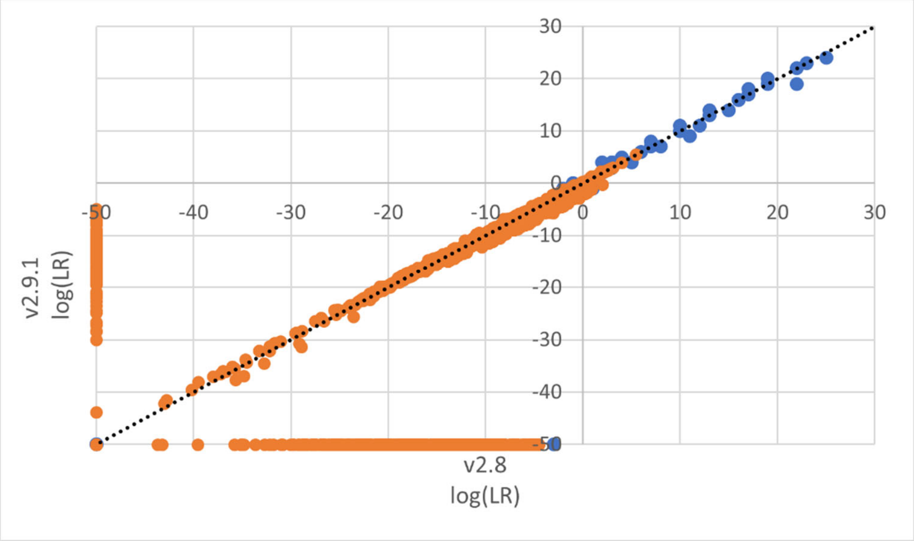

Supplementary Figure 2. Comparison of STRmixTM v2.8 and v2.9.1 (n=55)
